# Can Real-World Evidence Help Restore Decades of Health Inequalities by Informing Health Care Decision-Making? Certainly, and Here is How

**DOI:** 10.3389/fphar.2022.905820

**Published:** 2022-06-14

**Authors:** Grammati Sarri

**Affiliations:** Head of RWAA External Research Partnerships/Senior Research Principal, Cytel Inc., London, United Kingdom

**Keywords:** Real World Evidence (RWE), health disparities, value assessment, health technologies, novel methodologies

## Introduction

### Should we Pay Greater Attention to Health Conditions Unequally Distributed to Patient Groups who are Most Disadvantaged? How Should we Leverage Real-World Evidence (RWE) to Prioritise Scarce Resources to Reduce Health Inequalities?

**FIGURE 1:**
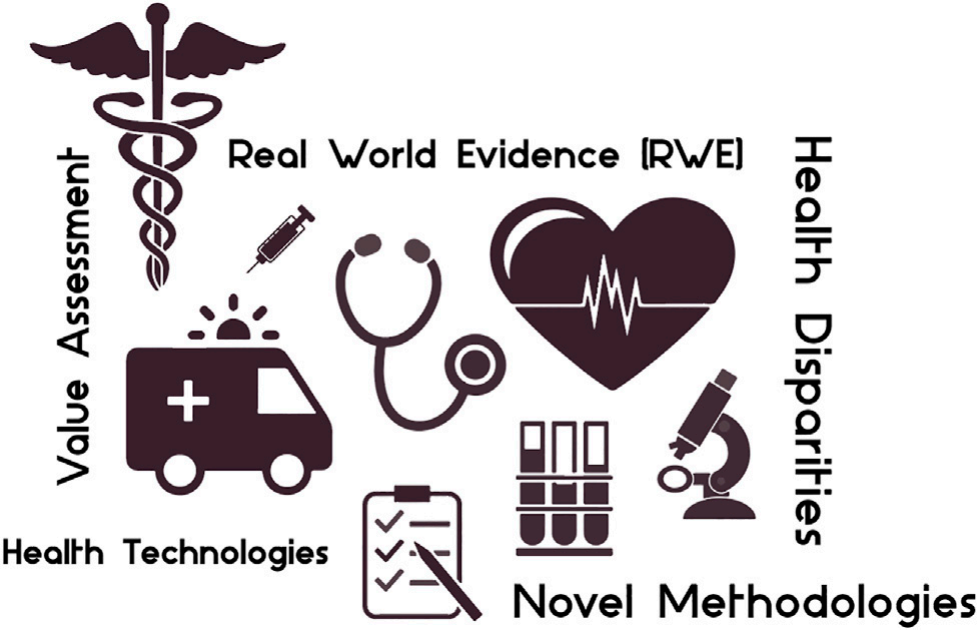
Drivers of healthcare decision-making process.

Attention to RWE generated outside traditional clinical trials has transformed the way evidence is collected and assessed when health technologies are appraised and decisions about healthcare are made (NICE, 2016; Arlett et al., 2022; Commissioner O of the. Real-World Evidence, 2022). The new era of digital health and big data analytics have further changed the type of evidence generated and shared with healthcare policy makers to inform their decisions (Stern et al., 2022). Several stakeholders have raised the profile of such evidence to fill in the gaps from traditional randomized clinical trials (RCTs) as a complementary evidence source. For example, RWE can provide comparative data (e.g establishing external controls) based on current standards of care when conducting RCTs is unethical or not feasible (e.g., for rare diseases with heterogenous patient populations not routinely enrolled in trials) (Chambers et al., 2016; Wu et al., 2020) or increasing the external validity of RCTs by offering a broader set of information (long-term effectiveness, tolerability of treatments in non-targeted populations in trials) for assessing the risk-benefit profile of technologies (Coleman et al., 2018; Peterson et al., 2019). However, scepticism about RWE data quality, transparency and the complexity of novel analytical methodologies have so far obstructed its wider use in decision-making, even though its significant impact on improving global health over the next couple of years has been widely recognised (World Health Organisation, 2022) (Figure 1).

Several developments and initiatives during recent years have contributed to fostering interest in RWE use for healthcare decision-making (Berger et al., 2017; Wang et al., 2017; Hampson et al., 2018; Gatto et al., 2019; Burcu et al., 2020; Sarri et al., 2020; Kent et al., 2021; Arlett et al., 2022). The COVID-19 pandemic has further demonstrated that reliance on clinical trials alone in assessments may delay access to novel, innovative health technologies (Franklin et al., 2021) and several opportunities arise from using evidence from RWE sources such as electronic health records (Sarri et al., 2022). However, using RWE comprehensively to capture sociodemographic heterogeneity from the patient’s experience especially for those most affected by the targeted disease and therefore most likely to be benefited by the technology under assessment, if efficacious and safe, has not been comprehensively considered so far in decision-making. The value and full potential of RWE to address health inequalities are still not fully recognised, leading to insufficient campaigning by stakeholders including patients for its wider consideration. This opinion paper will review and discuss if, how and when RWE has been previously used as a knowledge platform to argue unmet needs for disadvantaged patient groups and its impact in decision-making. Challenges and opportunities for further research in this area will also be explored.

### Why is RWE Needed in Technology Appraisals to Bridge the Health Inequality Gap?

Health inequalities are not a new public health issue: they still affect all countries around the globe to a varying degree (Organisation for Economic Co-operation and Development, 2019; Public Health Scotland, 2020; Health Inequalities, 2022) as has been shown through population or community RWE studies (Mahajan et al., 2021); however, what is new, is the large amount of real-world information on the heavy disease burden disproportionally experienced among the most disadvantaged in the society (Public Health England, 2020; Mishra et al., 2021). The unequal experience of good health and health gains is deeply rooted and well researched, from the well-defined social determinants of health to the access difficulties to appropriate healthcare and effective treatments (Marmot et al., 2012).

### How Often is Social Background Information Considered in the Trial Selection of Participants?

Let us start with how evidence is generated; when initial trials have established the safety of new technologies and promising effectiveness is indicated, a comprehensive clinical trial programme is launched with the aim to establish treatment’s efficacy and safety for regulatory and reimbursement submissions (FDA: Commissioner O of the Drug Development Process, 2020). These clinical trials are guided by strict study protocols with well-defined inclusion and exclusion criteria to ensure homogeneity of clinical trial participants. New health technologies are trialled in highly selected patient populations. It is well documented that the profile of patients recruited in clinical trials does not reflect the heterogeneous patient groups most likely to be recipients of these technologies when approved for use in routine clinical care. Previous research showed that trials primarily recruit affluent, younger, white male participants, leading ultimately to skewed data (Alegria et al., 2021). Marginalised communities may not be accounted for in algorithms which predict high participation rates in trials (Farmer et al., 2022). Furthermore, medication adherence, which has long been a spine in ensuring effective healthcare management, is a long-standing issue adversely affecting people from disadvantaged environments which is mainly driven by social challenges and issues around health literacy (Birch et al., 1343; Tavakoli et al., 2018). The inability of RCTs to identify and acknowledge the heterogeneity of patients experiencing a medical condition or a disease may limit the benefits of medical innovations, underestimate the variety of side effects of new technologies and, most importantly, may contribute to perpetuation of existing health inequalities as the targeted population of medical interventions is strictly reflective of those participating to the clinical programmes. During trial design, there is an opportunity for capturing different levels of disease severity, understanding issues of polypharmacy as experienced in real life (for example, among elderly patients (Moga et al., 2019)) and early identifying potential barriers in technology’s uptake (for example, digital divide for prisoners (Edge et al., 2020)) that may further enable a better representation of patient population most likely to use the technology and benefit from it when approved in clinical practice. So, is it more of a necessity supplementing RCT evidence from the real-world experience in clinical practice? The trial-centric approach in drug development means that patient experience and needs are less well understood; whereas the ability to identify and compare variations in patient outcomes is unfeasible. Therefore, inevitably, health care decision-makers make inferences about a drug’s use to the general targeted patient population by depending on potentially high-quality evidence generated from a very restricted population.

### How is Patient Generalisability Ensured if Patient Participation is not Reflective of the Patient Cohort Who Will Most Likely Receive the New Treatment in Clinical Practice?

There is always a tipped balance between internal and external validity of findings from the clinical trials; so far, decision-makers are engrossed on ensuring evidence supporting the clinical and cost-effectiveness (when applicable) of new health technologies is “free” from biases. But how does this exclusive focus impact on payers seeing the “complete” set of data to inform decision-making?

It is well known that poverty, lack of education and social exclusion have mostly contributed to creating an unequal burden in health wealth between the more and least advantaged members of our society. However, not much attention has been paid to how the current established system of trial-based evidence generation for new health technologies may not only be unable to address but even, in some cases, unintentionally exacerbate these health inequalities. The root problem in all of this lies on the fact that, as Prof Cookson rightly described, that decision makers have traditionally focused on the vertical axis when assessing new drugs: clinical and cost-effectiveness and impact on total population health. The horizontal axis is missing (impact on health equity). Recently, HTA bodies such as NICE in the UK have put health inequalities in the front line of their agenda^1^; however, as it is often the case when trying to quantify such a complex concept in decision-making, this was not formally considered as a modifier (weighting factor) in decision-making. Social inequalities can arise at different phases on the health intervention pathway with varying factors impacting on health inequality. There a spiral link between pre-existing health inequalities (before patients are unwell) and the inability of clinical trial programmes to capture the distribution of health benefits a technology may offer to patients due to differences in patient sociodemographic characteristics, barriers in the technology’s uptake and access difficulties. The current decision-making process also limits the opportunity to capture the full direction and magnitude of impact a new technology may have on health inequalities, allowing for the distribution of health opportunity costs as well as health benefits^2^. For example, this can be achieved by modifying the economic modelling structure and allowing the adjustment of utility values depending on the representation of disadvantaged groups with a particular disease or condition. Ultimately, questions on incorporating health inequalities in decision-making of new health technologies lie on the political will to address the equity-efficiency dilemma; whether it seems worth funding a treatment for patients who are currently severely ill even if no evidence has been requested if it reduces or, even worse, increases health inequalities or whether it is worth funding a preventive intervention that reduces health inequalities even if it benefits people who are not currently severely ill? How much can the cost-effectiveness modelling be modified to account for interventions with evidence of increase or reduction of health inequalities?

### So What Comes Next?

The world is changing; the pandemic, war and conflicts and the increasing poverty among patients already marginalised who are also more likely to suffer from poor health.

All the challenges anticipated due to financial and social pressures accumulated during recent years: disadvantaged groups are not only suffering from worse health with limited access to healthcare, but they are also most often underrepresented in health policies. This is the result of multiple interacting factors but also driven by the current process of decision-making which do not require health-related data to be representative of a wider, socially, and ethnically diverse patient evidence base.

Despite the challenges so far, and missed opportunities of the past, RWE research must move forward to build new research capacity for comprehensively capturing patient experience reflecting diversity in social and ethnic backgrounds. This can only come as a recognition of its potential and can be achieved through transparency, collaboration, and engagement at more holistic levels with patients, research organizations, industry, and decision makers. Recent examples of RWE large database-analyses provided information that can facilitate interventions to address existing health inequalities (Albertson et al., 2017; Piccinni et al., 2020; Piccinni et al., 2021). The RWE research community has undoubtedly a deep knowledge of challenges in RWE generation and analysis. However, researchers are now equipped with 21^st^ century digital and big data analytics tools to generate high-quality, fit-for-purpose RWE that can understand, account, and quantify for health inequalities and their impact on healthcare outcomes of new health technologies. Simultaneously, it is for decision-making bodies to consider how health inequalities can be captured in a structured and methodologically sound way during assessing the impact of new technologies. Contextual considerations such as social and environmental factors are already part of decision-making when assessing the value of new treatments although not always considered in a structured way and uncertain how their weight influence the final decisions^3^. The problem so far is that health inequality evidence is not consistently used to guide policy makers (Roldós and Breen, 2021). Expanding traditional cost effectiveness models (such as distributional cost-effectiveness analysis) can facilitate the ways to quantify health equality impacts and trade-offs in decision-making beyond the average health gain and losses of new technologies (Love- Koh et al., 2019). Additionally, it is important, although overlooked, that health related quality-of life data which are used to construct health outcome measures in decision-making (such as QALYs and utilities) should be collected in a reliable way to represent patient impact across populations experiencing health inequalities.

As previously noted, it is without doubt encouraging that, recently, HTA bodies have started recognising that health inequalities should no longer be ignored in decision-making for new technologies; in the UK, NICE encouraged further research on how health inequalities can be quantitatively accounted for (as another type of decision-modifier) in the clinical and cost-effectiveness assessment of new technologies. Moreover, RWE collection need also to prioritise diversity to reduce bias and maintain equity in patient representation; for example, previous studies have shown that increase in data from medical wearables only increase the gap between those with and without access to interconnected devices (Zhang et al., 2022; Scientific Reports, 2022). It is also encouraging that, FDA has just drafted detailed guidance to improve clinical trial diversity by explicitly requesting manufacturers to demonstrate that measures have been taken to enhance diversity in clinical trials (FDA, 2022). This can be done by collecting and analysing racial and ethnic data and broadening the trial eligibility criteria, when appropriate, to improve the patient representation affected by the targeted disease. Even though the specific guidance focuses on diversity arise by race and ethnicity, FDA recognises that underrepresentation in clinical trials may also arise from gender identity, age, pregnancy status and the presence of other conditions such as comorbidities.

To conclude, RWE has a great potential to reveal real life patient experiences and is a necessary channel to shed light on understanding and addressing health inequalities in health technology development and assessment. RWE can uncover not only heterogeneity in a technology’s clinical outcomes among patients in the real world but also identify barriers that can enable healthcare decision-makers to create a more equitable healthy society. This can only be done through ensuring diversity in data collection, for example better coding to capture sociodemographic data, but also increase in incentives for RWE infrastructure in deprived and marginalised communities. These efforts may formalise the processes to consider equity elements in data development life cycles. Including diverse populations in clinical research may lead to better, more robust data, greater equality, and, eventually, fewer disparities in health outcomes.

## Footnotes

1
https://www.nice.org.uk/Media/Default/About/what-we-do/our-programmes/nice-guidance/chte-methods-consultation/Equalities-task-and-finish-group-report.docx.

2
https://www.ispor.org/docs/default-source/euro2021/europe21kowal.pdf?sfvrsn=8c602e26_0.

3
https://journalofethics.ama-assn.org/sites/journalofethics.ama-assn.org/files/2021-07/stas1-peer-2108_0.pdf.

## References

[B1] Albertson T. E. Murin S. Sutter M. E. Chenoweth J. A. (2017). The Salford Lung Study: a Pioneering Comparative Effectiveness Approach to COPD and Asthma in Clinical Trials. Pragmat. Obs. Res. 8, 175–181. 10.2147/POR.S144157 29033625 PMC5614786

[B2] Alegria M. Sud S. Steinberg B. E. Gai N. Siddiqui A. (2021). Reporting of Participant Race, Sex, and Socioeconomic Status in Randomized Clinical Trials in General Medical Journals, 2015 vs 2019. JAMA Netw. Open 4 (5), e2111516. 10.1001/jamanetworkopen.2021.11516 34037736 PMC8155820

[B3] Arlett P. Kjaer J. Broich K. Cooke E. (2022). Real-World Evidence in EU Medicines Regulation: Enabling Use and Establishing Value. Clin. Pharmacol. Ther. 111 (1), 21–23. 10.1002/cpt.2479 34797920 PMC9299492

[B4] Berger M. L. Sox H. Willke R. J. Brixner D. L. Eichler H. G. Goettsch W. (2017). Good Practices for Real-World Data Studies of Treatment and/or Comparative Effectiveness: Recommendations from the Joint ISPOR-ISPE Special Task Force on Real-World Evidence in Health Care Decision Making. Pharmacoepidemiol Drug Saf. 26 (9), 1033–1039. 10.1002/pds.4297 28913966 PMC5639372

[B5] Birch J. M. Jones R. A. Mueller J. McDonald M. D. Richards R. Kelly M. P. . A Systematic Review of Inequalities in the Uptake of, Adherence to, and Effectiveness of Behavioral Weight Management Interventions in Adults. Obes. Rev. n/a, e13438. 10.1111/obr.13438 PMC9285567 35243743

[B6] Burcu M. Dreyer N. A. Franklin J. M. Blum M. D. Critchlow C. W. Perfetto E. M. (2020). Real-world Evidence to Support Regulatory Decision-Making for Medicines: Considerations for External Control Arms. Pharmacoepidemiol Drug Saf. 29 (10), 1228–1235. 10.1002/pds.4975 32162381 PMC7687199

[B7] Chambers D. A. Feero W. G. Khoury M. J. (2016). Convergence of Implementation Science, Precision Medicine, and the Learning Health Care System: A New Model for Biomedical Research. JAMA 315 (18), 1941–1942. 10.1001/jama.2016.3867 27163980 PMC5624312

[B8] Coleman C. I. Bunz T. J. Eriksson D. Meinecke A. K. Sood N. A. (2018). Effectiveness and Safety of Rivaroxaban vs Warfarin in People with Non-valvular Atrial Fibrillation and Diabetes: an Administrative Claims Database Analysis. Diabet. Med. 35 (8), 1105–1110. 10.1111/dme.13648 29663521

[B9] Commissioner O of the Real-World Evidence (2022). FDA. Available at: https://www.fda.gov/science-research/science-and-research-special-topics/real-world-evidence (Accessed on Jan 26, 2022).

[B13] Edge C. Hayward A. Whitfield A. Hard J. (2020). COVID-19: Digital Equivalence of Health Care in English Prisons. Lancet Digit. Health 2 (9), e450–2. 10.1016/S2589-7500(20)30164-3 32838249 PMC7377725

[B15] Farmer N. Osei Baah F. Williams F. Ortiz-Chapparo E. Mitchell V. M. Jackson L. (2022). Use of a Community Advisory Board to Build Equitable Algorithms for Participation in Clinical Trials: a Protocol Paper for HoPeNET. BMJ Health Care Inf. 29 (1), e100453. 10.1136/bmjhci-2021-100453 PMC8860013 35185011

[B10] FDA: Commissioner O of the Drug Development Process (2020). FDA. Available at: https://www.fda.gov/patients/learn-about-drug-and-device-approvals/drug-development-process (Accessed on Mar 24, 2022).

[B12] FDA. (2022). Diversity Plans to Improve Enrollment of Participants from Underrepresented Racial and Ethnic Populations in Clinical Trials; Draft Guidance for Industry. London: U.S. Food and Drug Administration. FDA, Available at: https://www.fda.gov/regulatory-information/search-fda-guidance-documents/diversity-plans-improve-enrollment-participants-underrepresented-racial-and-ethnic-populations (Accessed on Apr 29, 2022).

[B16] Franklin J. M. Lin K. J. Gatto N. M. Rassen J. A. Glynn R. J. Schneeweiss S. (2021). Real-World Evidence for Assessing Pharmaceutical Treatments in the Context of COVID-19. Clin. Pharmacol. Ther. 109 (4), 816–828. 10.1002/cpt.2185 33529354 PMC8014840

[B17] Gatto N. M. Reynolds R. F. Campbell U. B. (2019). A Structured Preapproval and Postapproval Comparative Study Design Framework to Generate Valid and Transparent Real-World Evidence for Regulatory Decisions. Clin. Pharmacol. Ther. 106 (1), 103–115. 10.1002/cpt.1480 31025311 PMC6771466

[B18] Hampson G. Towse A. Dreitlein W. B. Henshall C. Pearson S. D. (2018). Real-world Evidence for Coverage Decisions: Opportunities and Challenges. J. Comp. Eff. Res. 7 (12), 1133–1143. 10.2217/cer-2018-0066 30411972

[B19] Health Inequalities (2022). Oecd.Available at: https://www.oecd.org/health/inequalities-in-health.htm (Accessed on Mar 24, 2022).

[B20] Kent S. Salcher-Konrad M. Boccia S. Bouvy J. C. Waure C. Espin J. (2021). The Use of Nonrandomized Evidence to Estimate Treatment Effects in Health Technology Assessment. J. Comp. Eff. Res. 10 (14), 1035–1043. 10.2217/cer-2021-0108 34279114

[B21] Love-Koh J. Cookson R. Gutacker N. Patton T. Griffin S. (2019). Aggregate Distributional Cost-Effectiveness Analysis of Health Technologies. Value Health 22 (5), 518–526. 10.1016/j.jval.2019.03.006 31104729

[B22] Mahajan S. Caraballo C. Lu Y. Valero-Elizondo J. Massey D. Annapureddy A. R. (2021). Trends in Differences in Health Status and Health Care Access and Affordability by Race and Ethnicity in the United States, 1999-2018. JAMA 326 (7), 637–648. 10.1001/jama.2021.9907 34402830 PMC8371573

[B23] Marmot M. Allen J. Bell R. Bloomer E. Goldblatt P. (2012). WHO European Review of Social Determinants of Health and the Health Divide. Lancet 380 (9846), 1011–1029. 10.1016/S0140-6736(12)61228-8 22964159

[B24] Mishra V. Seyedzenouzi G. Almohtadi A. Chowdhury T. Khashkhusha A. Axiaq A. (2021). Health Inequalities during COVID-19 and Their Effects on Morbidity and Mortality. J. Healthc. Leadersh. Vol. 13, 19–26. 10.2147/jhl.s270175 PMC7826045 33500676

[B25] Moga D. C. Beech B. F. Abner E. L. Schmitt F. A. El Khouli R. H. Martinez A. I. (2019). INtervention for Cognitive Reserve Enhancement in Delaying the Onset of Alzheimer's Symptomatic Expression (INCREASE), a Randomized Controlled Trial: Rationale, Study Design, and Protocol. Trials 20, 806. 10.1186/s13063-019-3993-0 31888732 PMC6937673

[B27] Nice D. S. U. (2016). The Use of Real World Data for the Estimation of Treatment Effects in NICE Decision Making . https://www.google.com/search?q=NICE+DSU.+The+use+of+real+world+data+for+the+estimation+of+treatment+effects+in+NICE+decision+making%2C+2016.&rlz=1C1GCEA_enGB980GB980&oq=NICE+DSU.+The+use+of+real+world+data+for+the+estimation+of+treatment+effects+in+NICE+decision+making%2C+2016.&aqs=chrome.69i57j69i64.747j0j7&sourceid=chrome&ie=UTF-8 (Accessed on Feb 18, 2022).

[B28] Organisation for Economic Co-operation and Development (2019). Health for Everyone? Social Inequalities in Health and Health Systems (Paris: OECD OECD health policy studies), 187.

[B29] Peterson E. D. Ashton V. Chen Y. W. Wu B. Spyropoulos A. C. (2019). Comparative Effectiveness, Safety, and Costs of Rivaroxaban and Warfarin Among Morbidly Obese Patients with Atrial Fibrillation. Am. Heart J. 212, 113–119. 10.1016/j.ahj.2019.02.001 30981035

[B30] Piccinni C. Cevoli S. Ronconi G. Dondi L. Calabria S. Pedrini A. (2021). Insights into Real-World Treatment of Cluster Headache through a Large Italian Database: Prevalence, Prescription Patterns, and Costs. Expert Rev. Clin. Pharmacol. 14 (9), 1165–1171. 10.1080/17512433.2021.1934448 34030566

[B31] Piccinni C. Dondi L. Ronconi G. Calabria S. Esposito I. Pedrini A. (2020). Real-world Data on New Users of Atypical Antipsychotics: Characterisation, Prescription Patterns, Healthcare Costs and Early Cardio-Metabolic Occurrences from a Large Italian Database. Eur. J. Clin. Pharmacol. 76 (9), 1301–1310. 10.1007/s00228-020-02899-9 32462326

[B11] Public Health England (2020). Disparities in the Risk and Outcomes of COVID-19. London: Public Health England.

[B38] Public Health Scotland (2020). What Are Health Inequalities? London: The King’s Fund. (Accessed on Mar 24, 2022). Available at: https://www.kingsfund.org.uk/publications/what-are-health-inequalities.

[B32] Roldós M. I. Breen N. (2021). Using Economic Evaluation to Hasten Health Equity. Health Equity 5 (1), 627–632. Available at: https://www.liebertpub.com/doi/full/10.1089/heq.2021.0010 . 34909530 10.1089/heq.2021.0010 PMC8665794

[B33] Sarri G. Bennett D. Debray T. Deruaz-Luyet A. Soriano Gabarró M. Largent J. A. (2022). ISPE-endorsed Guidance in Using Electronic Health Records for Comparative Effectiveness Research in COVID-19: Opportunities and Trade-Offs. Clin. Pharmacol. Ther. 10.1002. Available at: https://onlinelibrary.wiley.com/doi/abs/10.1002/cpt.2560 . 10.1002/cpt.2560 PMC9087010 35170021

[B34] Sarri G. Patorno E. Yuan H. Guo J. Jeff) Bennett D. (2020). Framework for the Synthesis of Non-randomised Studies and Randomised Controlled Trials: a Guidance on Conducting a Systematic Review and Meta-Analysis for Healthcare Decision Making. BMJ Evid-Based Med. 27, 109–119. Available at: https://ebm.bmj.com/content/early/2020/12/09/bmjebm-2020-111493 . 10.1136/bmjebm-2020-111493 PMC8961747 33298465

[B26] Scientific Reports (2022). A National Survey Assessing Public Readiness for Digital Health Strategies against COVID-19 within the United Kingdom. Available at: https://www.nature.com/articles/s41598-021-85514-w (Accessed on Mar 24, 2022). 10.1038/s41598-021-85514-w PMC7966397 33727655

[B35] Stern A. D. Brönneke J. Debatin J. F. Hagen J. Matthies H. M. Patel S. (2022). Advancing Digital Health Applications: Priorities for Innovation in Real-World Evidence Generation. Lancet Digit. Health 4 (3), e200–6. 10.1016/S2589-7500(21)00292-2 35216754

[B36] Tavakoli H. Mark FitzGerald J. Lynd L. D. Sadatsafavi M. (2018). Predictors of Inappropriate and Excessive Use of Reliever Medications in Asthma: a 16-year Population-Based Study. BMC Pulm. Med. 18 (1), 33. 10.1186/s12890-018-0598-4 29433489 PMC5809893

[B37] Wang S. V. Schneeweiss S. Berger M. L. Brown J. de Vries F. Douglas I. (2017). Reporting to Improve Reproducibility and Facilitate Validity Assessment for Healthcare Database Studies V1.0. Value Health 20 (9), 1009–1022. 10.1016/j.jval.2017.08.3018 28964431

[B14] World Health Organisation. (2022). Emerging Trends and Technologies: A Horizon Scan for Global Public Health Available at: https://www.who.int/publications-detail-redirect/9789240044173 (Accessed on Mar 24, 2022).

[B39] Wu J. Wang C. Toh S. Pisa F. E. Bauer L. (2020). Use of Real-World Evidence in Regulatory Decisions for Rare Diseases in the United States-Current Status and Future Directions. Pharmacoepidemiol Drug Saf. 29 (10), 1213–1218. 10.1002/pds.4962 32003065

[B40] Zhang J. Symons J. Agapow P. Teo J. T. Paxton C. A. Abdi J. (2022). Best Practices in the Real-World Data Life Cycle. PLOS Digit. Health 1 (1), e0000003. 10.1371/journal.pdig.0000003 PMC9931348 36812509

